# The Meeting of the Georgia Medical Association

**Published:** 1902-04

**Authors:** 


### THE MEETING OF THE GEORGIA MEDICAL ASSOCIATION.

ELSEWHERE may be found the preliminary program of the Savannah meeting of the State Medical Association. In the large number of topics to be discussed there is sure to be something that will interest each member and make his attendance upon the meeting a source of profit as well as of pleasure. The reputation that Savannah has always enjoyed for hospitality and cordial greeting will be an additional inducement for the members to lay aside for a few days the burden of medical practice and enjoy the peculiar diversion and pleasure of friendly association with their fellow craftsmen. The meeting promises to be well attended and it is hoped that this promise will be abundantly fulfilled and that every one will return a more enthusiastic friend of the Association and more than ever in love with his profession.

